# Recent advances in Mpox across innate immunity, evasion, and clinical outcomes

**DOI:** 10.1038/s42003-026-10487-3

**Published:** 2026-06-15

**Authors:** Jueun Oh, Yoon-Seok Chung, SangJoon Lee

**Affiliations:** 1 Department of Biological Science, Ulsan National Institute of Science and Technology (UNIST), Ulsan, Republic of Korea; 2 Division of Acute Viral Diseases, Center for Emerging Virus Research, National Institute of Infectious Diseases, Korea National Institute of Health, Cheongju, Republic of Korea; 3 Graduate School of Health Science and Technology, Ulsan National Institute of Science and Technology (UNIST), Ulsan, Republic of Korea

**Keywords:** NOD-like receptors, Viral infection

## Abstract

Recent mpox outbreaks have shown that disease severity cannot be explained by viral burden alone but is instead closely linked to the host immune response. This review synthesizes current evidence on the genomic diversity of monkeypox virus (MPXV), innate immune sensing, inflammatory pathways, immune evasion, and therapeutic strategies. We examine the key host determinants of disease outcome, including interferon signaling competence, inflammasome regulation, and natural killer cell function, together with the clinical manifestations seen in immunocompromised populations. By integrating molecular and clinical perspectives, this review helps explain the heterogeneity of mpox and informs the management of high-risk populations.

## Abstract (web summary)

This review explains how the balance between innate immune sensing and viral immune evasion shapes the severity of mpox, linking molecular mechanisms of monkeypox virus infection to the varied clinical outcomes seen in recent outbreaks.

## Introduction

In contrast to earlier episodes of mpox that were predominantly zoonotic and geographically constrained, the 2022 epidemic was driven by Clade IIb lineages that achieved efficient human-to-human transmission across multiple regions and transmission networks^1–3^. The clinical phenotype during this period was characterized by prominent mucocutaneous and anogenital lesions, prolonged detection of viral DNA at mucosal sites, and substantial inter-individual heterogeneity in the disease course^1,2^. This combination of sustained transmission and variable clinical trajectories indicates that early host-virus interactions contribute to viral containment and inflammatory tissue injury underlying clinical complications^4^. Recent clinical data indicate that mpox severity cannot be predicted by viral burden, particularly in patients with extensive mucocutaneous involvement, necrotic lesions, or systemic complications^1,5^. This perspective is clinically relevant because severe disease is enriched in individuals with functional immunodeficiency, including those with advanced human immunodeficiency virus (HIV) infection, wherein prolonged viral replication and dysregulated inflammation may coexist and contribute to morbidity^5,6^.

Similar to other DNA viruses, MPXV engages cytosolic DNA-sensing pathways that shape antiviral restriction and inflammatory pathology. The cGAS-STING axis contributes to Type I IFN induction and viral control^7^, whereas the AIM2 inflammasome links viral DNA sensing to caspase-1 activation, IL-1β and IL-18 release, and inflammatory cell death^8,9^. Together, these pathways reflect the balance between viral containment and tissue injury, whose relative contributions vary across cell types, tissue contexts, and host immune states. MPXV also encodes immunomodulatory factors that target host defenses at multiple nodes. Reported mechanisms include interference with signal propagation in cGAS-STING-TANK-binding kinase 1 (TBK1)-interferon regulatory factor 3 (IRF3) signaling, suppression of IRF3 nuclear accumulation, extracellular antagonism of Type I IFN activity, and inhibition of signal transducer and activator of transcription 2 (STAT2)-dependent transcriptional outputs downstream of IFN receptors^10–14^. Additional viral interfaces that affect the complement, tumor necrosis factor (TNF) signaling, and co-stimulatory pathways demonstrate that innate activation during infection reflects the interplay between host-recognition pathways and viral modulation of antiviral and inflammatory responses^15–19^.

This review synthesizes evidence linking MPXV genomic diversity, innate immune sensing, immune evasion, clinical immunopathogenesis, and therapeutic strategies to disease heterogeneity, with emphasis on host immune determinants of clinical outcome.

### Genomic organization, evolution, and clade diversity of MPXV

Mpox is a zoonotic viral disease caused by MPXV^20^. MPXV is a member of the genus *Orthopoxvirus* within the family *Poxviridae*, alongside Variola virus (VARV), Cowpox virus (CPXV), Vaccinia virus (VACV), and Ectromelia virus (ECTV). MPXV is an enveloped, brick-shaped virus with a linear double-stranded DNA genome of approximately 197 kb^21^.

Structurally, the genome is organized into highly conserved central and variable terminal regions^22,23^. The terminal regions contain inverted terminal repeats (ITRs) and are enriched in genes involved in host immune evasion, host-range determination, and virulence regulation^24^. Recent studies on MPXV genomes have demonstrated rapid structural remodeling at their termini through genomic accordion-type processes. Length variations and rearrangements in low-complexity regions along with single-nucleotide polymorphisms (SNPs) can contribute substantially to the differences between isolates^25,26^. Furthermore, isolates collected during the 2022–2023 outbreak exhibited extensive terminal gene loss and the emergence of novel open reading frames (ORFs) as a result of unique deletions ranging from 573 to 21,576 bp^27^. MPXV strains are phylogenetically divided into Clade I (Central African) and Clade II (West African), which differ in geographic origin and disease severity^28,29^. The case fatality rate has been reported to be approximately 10.6% for Clade I and 3.6% for Clade II^1,30^. The 2022 global outbreak was driven by Clade IIb, which acquired sustained human-to-human transmission capabilities and was presumed to have diverged from Clade IIa^3^. More recently, Clade Ib, a lineage within Clade I characterized by increased human transmissibility, has been reported to be an emerging public health threat^31–33^. Genetic differences between clades were primarily observed in terminal genes. For instance, the *D14L* gene, which encodes the monkeypox inhibitor of complement enzymes (MOPICE), is present in Clade I genomes but is absent from Clade II lineages^17,34^. Notably, an extensive apolipoprotein B mRNA editing catalytic polypeptide-like 3 (APOBEC3)-mediated mutational signature has been observed in MPXV genomes isolated since 2017, particularly following the 2022 outbreak^35–39^. Clade IIb and Ib genomes exhibited a high accumulation of point mutations in the form of TC to TT or GA to AA, consistent with editing by the host antiviral enzyme APOBEC3^32,33,35,36^. This specific mutational pattern is rarely found in sporadic zoonotic cases but appears selectively in lineages undergoing sustained human-to-human transmission^38^. These patterns support ongoing MPXV microevolution during sustained human-to-human transmission.

### Overview of host innate immunity to MPXV

Host defense against MPXV involves cytosolic DNA sensing, interferon-mediated restriction, inflammasome-mediated inflammatory execution, and in vivo determinants of disease outcome. The relative contribution of these processes varies by cell type, tissue context, and host immune state.

### Cytosolic DNA sensing and IFN

**Fig. 1:**
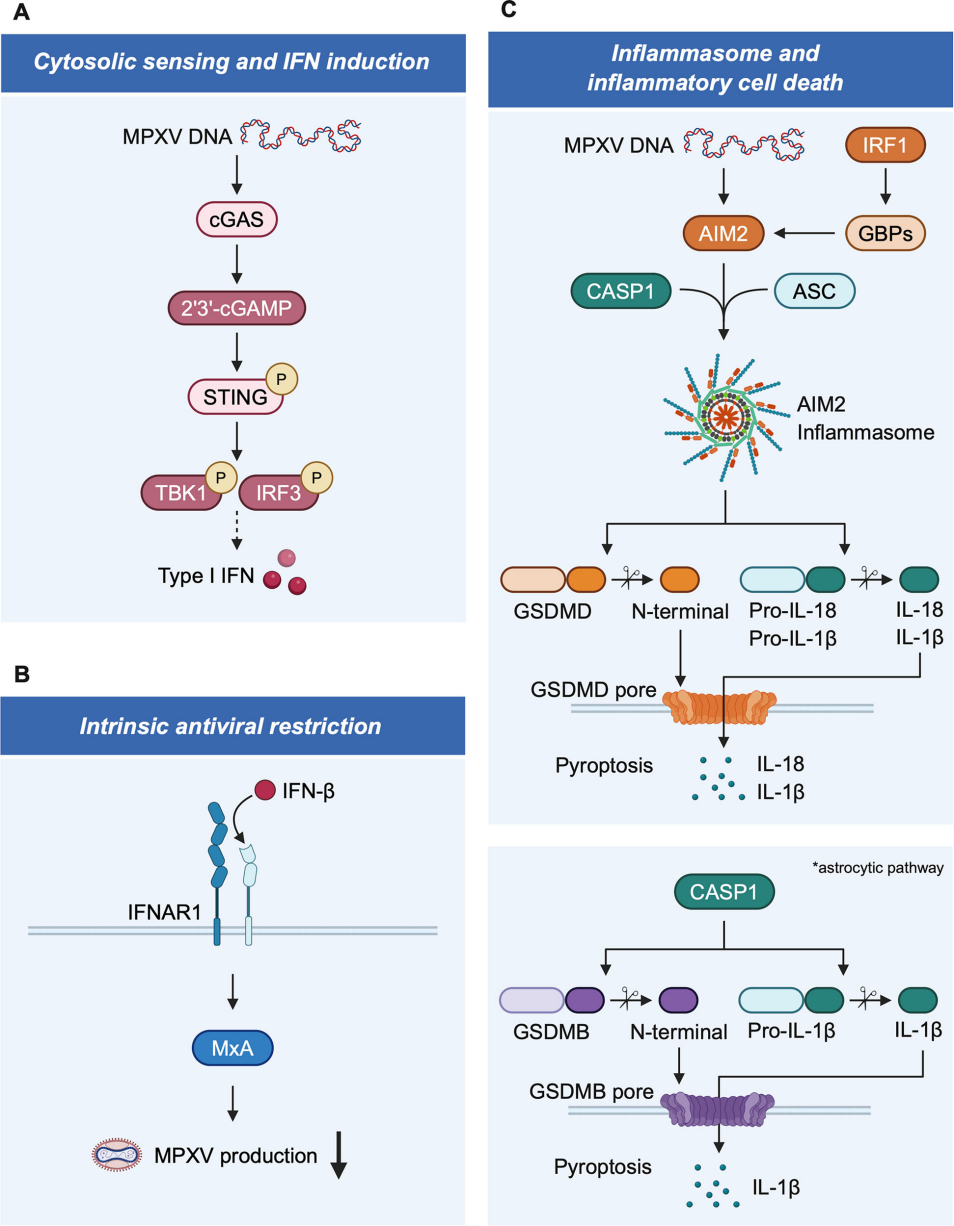
Innate immune responses to MPXV. **A** Cytosolic MPXV DNA is sensed by cGAS, leading to 2′3′-cGAMP production, STING phosphorylation, and downstream TBK1 and IRF3 phosphorylation, culminating in Type I IFN production. **B** IFN-β signals through IFNAR1 to induce MxA, which is associated with reduced MPXV production. **C** MPXV DNA activates the AIM2 inflammasome through IRF1- and GBP-dependent priming. AIM2-ASC complex assembly recruits and activates CASP1, which cleaves GSDMD to form membrane pores and processes Pro-IL-18 and Pro-IL-1β into mature IL-18 and IL-1β, driving pyroptosis and cytokine release. In human astrocytes (asterisk), CASP1 instead cleaves GSDMB, forming GSDMB pores and driving pyroptosis with IL-1β release. AIM2 absent in melanoma 2, ASC apoptosis-associated speck-like protein containing a CARD, CASP1 caspase-1, cGAMP cyclic GMP-AMP, cGAS cyclic GMP-AMP synthase, GBPs guanylate-binding proteins, GSDMB gasdermin B, GSDMD gasdermin D, IFN interferon, IFNAR1 interferon-α/β receptor subunit 1, IL interleukin, IRF interferon regulatory factor, MPXV monkeypox virus, MxA myxovirus resistance protein A, STING stimulator of interferon genes, TBK1 TANK-binding kinase 1. Created in BioRender. Oh, J. (2026) https://BioRender.com/7c2xvbe.

As a cytosolic DNA virus, MPXV engages DNA-sensing pathways, causing the induction of Type I IFN^4^. In a CAST/EiJ mouse model infected with MPXV Clade IIb, infection activated the cGAS-STING axis in myeloid cells. In infected bone marrow-derived macrophages (BMDMs), phosphorylation of STING, TBK1, and IRF3 occurred early after infection, with increased IFN-β transcription and IFN-stimulated gene (ISG) expression^7^ (Fig. 1A).

In human cells, productive MPXV infection was associated with limited endogenous Type I IFN transcription. Primary human macrophages, primary human fibroblasts, and HeLa cells showed weak IFN-associated signatures, whereas gamma-irradiated MPXV induced stronger IFN-associated transcription under comparable conditions^40^. In a multiomics study of MPXV-infected primary human foreskin fibroblasts, host responses were characterized by a proinflammatory signature with minimal induction of canonical Type I IFNs and ISGs within the profiled time course^41^. These findings indicate that IFN-related outputs depend on the experimental system and require direct pathway assessment^40,41^.

### Intrinsic antiviral restriction

IFN-β pretreatment and early post-infection treatment reduced infectious MPXV yields in human cells, with cell-type-dependent effects observed in primary fibroblasts and HeLa cells. IFN-β also induced MxA, and constitutive MxA expression reduced MPXV production, with partial co-localization between MxA and the viral protein A33^42^ (Fig. 1B).

Compared with VACV, MPXV produced lower dsRNA accumulation and minimal activation of the dsRNA-dependent protein kinase PKR, indicating weak engagement of canonical dsRNA-sensing stress responses under the tested conditions^43^.

Across tissue-relevant epithelial models, immune outputs did not consistently scale with productive MPXV replication. Human keratinocytes showed altered host pathways, including Toll-like receptor signaling, whereas lung organoids supported replication without major inflammatory mediator or IFN-β changes^44,45^. Kidney organoids showed MPXV-induced injury with antiviral responsiveness^46^. Overall, experimentally imposed Type I IFN states restricted MPXV through ISG-linked effectors such as MxA^42^, whereas endogenous IFN-associated transcription and dsRNA-PKR activation were limited during productive infection in multiple reported human cell systems^40,41,43^.

### Inflammasome and inflammatory cell death

MPXV infection can engage inflammatory execution pathways. This inflammatory response is dominated by the AIM2 inflammasome in myeloid cells, which serves as a critical cytosolic DNA sensor for various pathogens, including herpes simplex virus 1 (HSV-1)^47,48^. Clustered regularly interspaced short palindromic repeat (CRISPR)-based sensor screening and genetic validation identified AIM2 as the key inflammasome sensor required for MPXV-induced caspase-1 activation and IL-1β/IL-18 secretion in primary macrophages^8,9^. Disruption of canonical inflammasome components or gasdermin D (GSDMD) reduced cytokine release and lytic death, supporting the AIM2-ASC-caspase-1-GSDMD axis in macrophages^8,9^. Pyroptosis was preferentially observed in infected macrophages, whereas uninfected bystander cells displayed enrichment of apoptosis and necroptosis-signaling markers, supporting a mixed-death landscape that can amplify local inflammatory injury beyond the initially infected compartment^8,9,49–51^. Mechanistically, AIM2 activation during MPXV infection depends on IFN-linked priming and licensing. Interferon regulatory factor 1 (IRF1) is induced downstream of Type I IFN signaling and supports AIM2-dependent inflammasome responses during MPXV infection, and transcriptomic profiles have shown the induction of multiple GBP family members as part of a broader IFN-stimulated response accompanying this inflammatory state^8,9^ (Fig. 1C).

Notably, the cytopathic effects of MPXV are strongly cell-type-dependent, indicating that the innate inflammatory response is shaped by tissue context. In in vitro assessments, primary human astrocytes were shown to support productive MPXV infection and induce a lytic inflammatory death pathway consistent with pyroptosis, with gasdermin B (GSDMB), rather than GSDMD, acting as the terminal gasdermin effector^52^. Infected astrocytes upregulate inflammatory and inflammasome-related genes, including caspase-1 and IL-1β, and undergo proteolytic processing of GSDMB. This was accompanied by membrane-rupture phenotypes, supporting lytic inflammatory death in this central nervous system-relevant cell type^52^ (Fig. 1C). Importantly, this astrocytic pathway is mediated through GSDMB rather than the canonical GSDMD axis, which predominates in many myeloid inflammasome settings. GSDMB is expressed in humans but not in mice, highlighting the potential species- and tissue-specific constraints in extrapolating inflammatory execution pathways from standard animal models. These findings support a human-relevant, cell-type-specific inflammatory execution mechanism that may not be captured in standard mouse models^52^.

### Innate immune determinants of MPXV outcomes

MPXV outcomes vary across hosts and experimental systems, reflecting differences in genetic susceptibility, interferon signaling, inflammatory execution, natural killer (NK) cell function, and infection context.

#### Host and contextual determinants of susceptibility

**Fig. 2:**
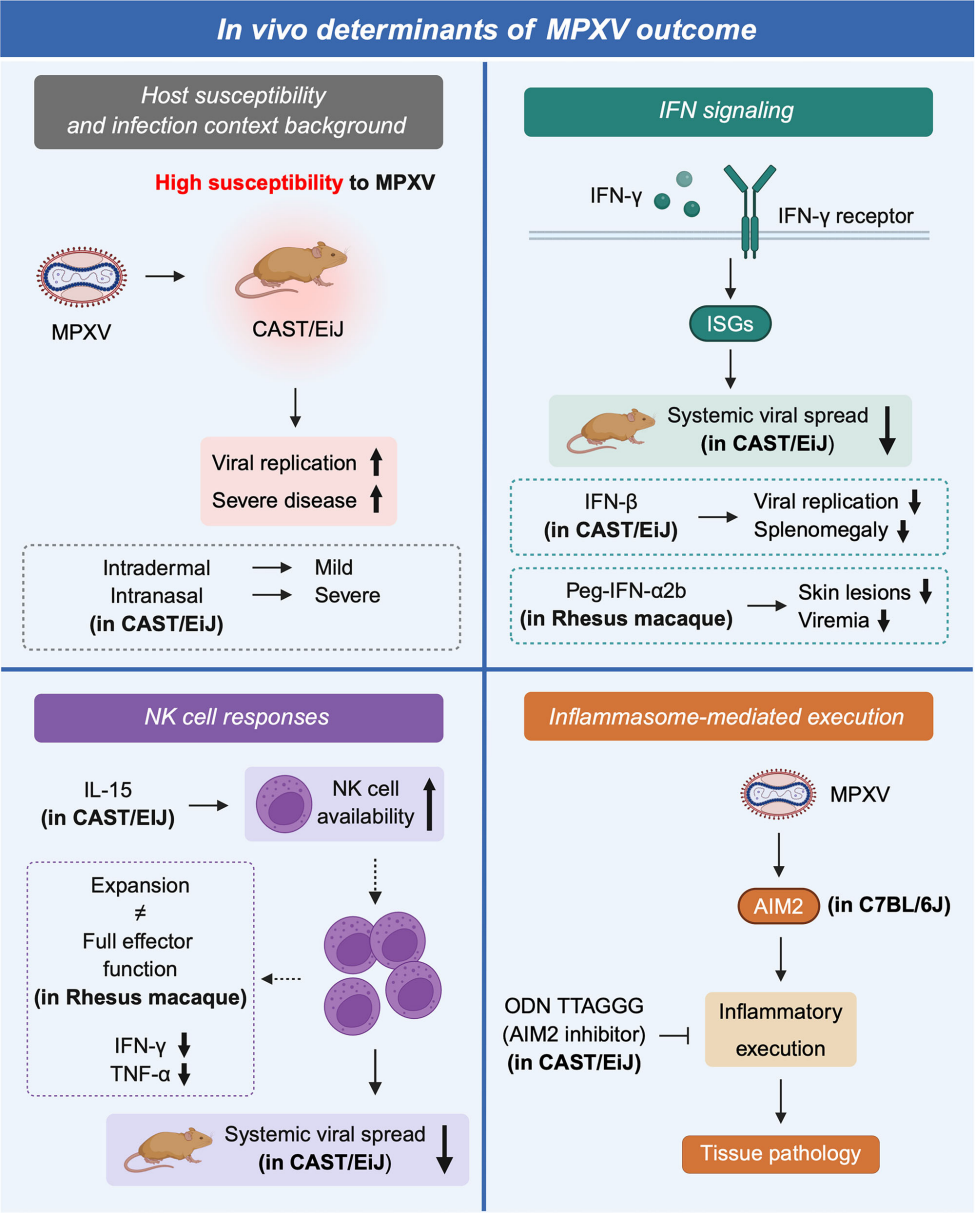
Host innate immune determinants of MPXV outcome. CAST/EiJ mice display high susceptibility to MPXV, with enhanced viral replication and severe disease. In CAST/EiJ mice, intradermal infection produces milder disease, whereas intranasal infection results in severe disease. IFN-γ signaling through the IFN-γ receptor induces ISGs and limits systemic viral spread in CAST/EiJ mice. Exogenous IFN-β administration reduced viral replication and splenomegaly in CAST/EiJ mice, whereas pegylated IFN-α2b reduced skin lesions and viremia in rhesus macaques. AIM2-dependent inflammatory execution, as characterized in C57BL/6 J mice, drives tissue pathology, and intraperitoneal administration of the AIM2 inhibitor ODN TTAGGG in CAST/EiJ mice reduced inflammatory execution and improved survival. Short-course IL-15 treatment in CAST/EiJ mice increased NK cell availability and reduced systemic viral spread. NK cell expansion in rhesus macaques was not accompanied by full effector function, as evidenced by reduced IFN-γ and TNF-α production.AIM2, absent in melanoma 2; IFN, interferon, IL interleukin, ISGs interferon-stimulated genes, MPXV monkeypox virus, NK natural killer, ODN oligodeoxynucleotide, TNF tumor necrosis factor. Created in BioRender. Oh, J. (2026) https://BioRender.com/81hm7ri.

Experimental infection studies in animal models have indicated that MPXV disease outcomes in mice vary according to host genetic background. In a multi-strain comparison, several wild-derived inbred strains exhibited markedly increased susceptibility in comparison with classical laboratory strains. Among these, CAST/EiJ mice that underwent intranasal MPXV infection consistently developed severe disease characterized by weight loss, systemic viral dissemination, and replication in multiple organs^53^ (Fig. 2). These findings establish host genetic background as a determinant of MPXV outcomes in murine models. Among nonhuman primate models, the marmoset *Callithrix jacchus* was shown to be susceptible to MPXV. Subcutaneous infection in a low-dose model led to severe disease; this approach has been used to evaluate the pathogenesis and therapeutic interventions^54^.

In addition to strain-dependent susceptibility, the infection route is also associated with the outcomes in CAST/EiJ mice. In an intradermal infection model using tail scarification in CAST/EiJ mice, Clade IIb MPXV induced milder disease than Clade IIa. This difference was associated with stronger and earlier induction of neutralizing antibody responses and Type I IFN during Clade IIb infection^55^. In cynomolgus macaques, route of exposure altered disease kinetics, with intrabronchial inoculation producing a more prolonged course than intravenous challenge, including delayed fever, lesions, viremia, shedding, and cytokine peaks^56^.

#### IFN signaling

In CAST/EiJ mice, administration of recombinant IFN-β reduced viral replication and alleviated splenomegaly. In rhesus macaques, pegylated IFN-α2b similarly reduced disease severity and plasma viremia. Together, these findings indicate that augmentation of Type I IFN signaling can constrain the systemic viral burden and modulate MPXV disease progression^7^.

Comparative in vivo profiling indicates that IFN-γ and CCL5 induction were absent in infected lung tissue of CAST/EiJ mice, in contrast to that in BALB/c mice, where both were robustly induced^57^. Intranasal delivery of recombinant IFN-γ provides protection from otherwise lethal MPXV challenge and reduces the lung viral burden. Complementary genetic loss-of-function experiments in C57BL/6 mice confirmed this relationship, as inactivation of IFN-γ or the IFN-γ receptor increased susceptibility to MPXV in this otherwise resistant background^57^ (Fig. 2).

#### Inflammasome-mediated execution

In vivo studies have indicated that AIM2-dependent inflammation can contribute to viral restriction while driving pathology when excessive^8^. In genetic loss-of-function settings, AIM2 deficiency reduces inflammasome-linked inflammatory outputs and leukocyte migration signatures but allows increased viral spread^8^. In susceptible CAST/EiJ mice, MPXV challenge produces rapidly lethal disease with severe tissue necrosis and systemic inflammation, and intraperitoneal administration of the AIM2 inhibitor (ODN TTAGGG) has been shown to improve survival with reduced IL-1β and IL-18 levels and tissue pathology^8^ (Fig. 2).

#### NK cell responses

In a rhesus macaque model of MPXV infection, the NK cell compartment expanded quantitatively during the acute phase. However, this increase was accompanied by functional impairment^58^. Ex vivo functional profiling demonstrated reduced degranulation and near-complete ablation of IFN-γ and TNF-α production across NK subsets, indicating that numerical expansion does not necessarily preserve effector competence in vivo^58^ (Fig. 2).

The functional contribution of NK cells was examined in a susceptible CAST/EiJ model. Short-course IL-15 administration increased NK cell numbers and generated populations capable of IFN-γ production upon stimulation^59^. Although NK cell counts declined after cessation of IL-15 treatment, IL-15 pretreatment was associated with reduced whole-body bioluminescent viral dissemination and improved survival following infection with luciferase-tagged MPXV^59^ (Fig. 2). Taken together, these data suggest that functional NK cell competence, and not cell number alone, is a relevant parameter for viral control in the studied preclinical models.

### Immune evasion of MPXV

MPXV immune evasion can be organized across nucleic acid sensing, Type I IFN induction, receptor-proximal signaling, and downstream transcriptional control^10–14^. Reported mechanisms include impaired IRF3 nuclear translocation^11^, dampened cGAS-STING signaling^12^, extracellular Type I IFN antagonism^13^, STAT2-dependent suppression of ISG transcription^14^, a tripartite motif-containing protein 5 alpha (TRIM5α)-associated axis^15^, complement modulation^16,17^, TNF antagonism^18^, and interference with B7-dependent co-stimulation^19^.

MPXV *F3L* is a truncated homolog of VACV *E3L* that lacks an N-terminal Z-DNA-binding domain. However, MPXV carrying naturally truncated *F3L* did not recapitulate the IFN-sensitive phenotype observed in analogous VACV truncation models, suggesting compensation by other MPXV genetic determinants^10^.

**Fig. 3:**
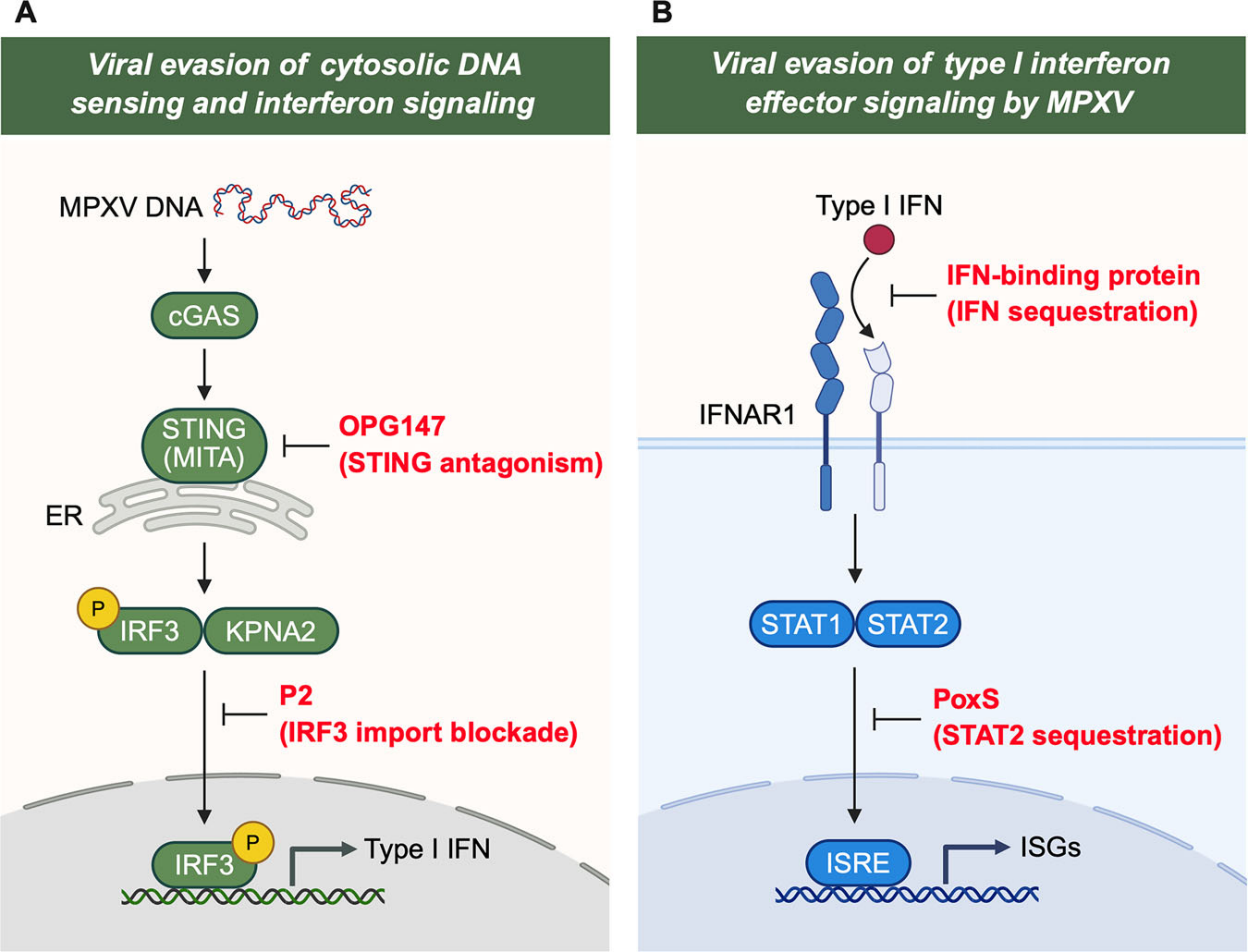
Immune evasion of MPXV. **A** MPXV antagonizes cytosolic DNA sensing and Type I IFN induction at the cGAS-STING-IRF3 axis. OPG147 targets STING (MITA) at the endoplasmic reticulum (ER) to inhibit STING activation and trafficking. P2 hijacks KPNA2 to block IRF3 nuclear import, reducing IRF3-dependent Type I IFN transcription. **B** MPXV suppresses Type I IFN effector signaling through an extracellular IFN-binding protein (IFNα/βBP) that sequesters circulating Type I IFN before receptor engagement at IFNAR1, and through PoxS, which sequesters STAT2 in the cytoplasm to reduce ISRE-linked ISG transcription. cGAS cyclic GMP-AMP synthase, ER endoplasmic reticulum, IFN interferon, IFNα/βBP type I interferon-binding protein, IFNAR1, interferon-α/β receptor subunit 1, IRF3 interferon regulatory factor 3, ISGs interferon-stimulated genes, ISRE interferon-stimulated response element, KPNA2 karyopherin subunit alpha 2, MITA/STING stimulator of interferon genes, MPXV monkeypox virus, PoxS poxin-schlafen fusion protein, STAT signal transducer and activator of transcription.Created in BioRender. Oh, J. (2026) https://BioRender.com/o6wf94e.

In cell-based assays, MPXV P2 suppressed IRF3-dependent Type I IFN production, primarily by limiting IRF3 nuclear accumulation, resulting in reduced *IFNB1*, *ISG15*, and *ISG20* mRNA levels. Notably, upstream readouts, including cGAS, STING, TBK1, and IRF3 abundance; TBK1 and IRF3 phosphorylation; and IRF3 dimerization were largely preserved^11^. Mechanistically, P2 contains a functional nuclear localization signal near the C-terminus that competitively hijacks Karyopherin subunit alpha 2 (KPNA2), thereby blocking the KPNA2-mediated IRF3 nuclear import. NLS deletion or mutation abrogated KPNA2 binding and restored IRF3 nuclear accumulation, together with *IFNB1*/*ISG* induction^11^ (Fig. 3A).

In parallel, MPXV OPG147 was identified as a conserved antagonist of the cGAS-MITA/STING pathway. OPG147 associates with MITA/STING in the endoplasmic reticulum and acts downstream of ligand sensing. Specifically, OPG147 disrupts MITA/STING ISGylation, higher-order assembly, and stimulus-induced trafficking to perinuclear signaling compartments^12^. These effects are associated with reduced recruitment of TBK1 and IRF3 to the signaling complex and with reduced levels of STING-responsive transcripts, including *IFNB1*, *CXCL10*, and *IL6*, in cell-based assays. In human myeloid cells, gRNA-mediated targeting of OPG147 enhances STING pathway activation during MPXV infection and decreases the production of infectious progeny^12^ (Fig. 3A).

MPXV encodes a secreted Type I IFN-binding protein (IFNα/βBP) that can associate with the cell surface after release. Supernatants containing IFNα/βBP reduced Type I IFN-induced STAT1 phosphorylation. In addition, pre-incubation of Type I IFNs with these supernatants neutralized their antiviral activity against the vesicular stomatitis virus (VSV) challenge in a dose-dependent manner^13^. The inhibitor was detected by immunoblotting the supernatants from MPXV-infected cells, confirming its active expression during infection. Notably, the MPXV IFNα/βBP bound human Type I IFNs but lacked detectable binding to murine IFNβ and Type III IFNs, a binding profile distinct from its VACV ortholog and consistent with adaptation toward the human IFN system^13^ (Fig. 3B). Furthermore, VARV encodes a structurally related IFN-binding protein that antagonizes Type I IFN. The human-specific binding profile of MPXV IFNα/βBP thus represents a convergent but host-adapted strategy shared across highly virulent orthopoxviruses^13^.

Downstream of the IFN receptors, MPXV encodes a poxin-Schlafen fusion protein (PoxS) that interacts with STAT2 and sequesters it in the cytoplasm. Notably, PoxS does not suppress cGAS-STING-mediated Type I IFN production; rather, it selectively inhibits downstream ISG transcription, including that of *OAS1*, *SAMD9*, *SAMD9L*, *ISG15*, *ISG56*, and *IFIT3*, by blocking basal and Type I IFN-induced ISRE-linked transcriptional outputs^14^. Both the Schlafen fusion domain and the catalytic site of the 2′3′-cGAMP nuclease are required for this activity, as demonstrated by suppression of ISRE activity and reduced sensitivity to Type I IFN-mediated restriction of MPXV replication^14^ (Fig. 3B).

**Fig. 4:**
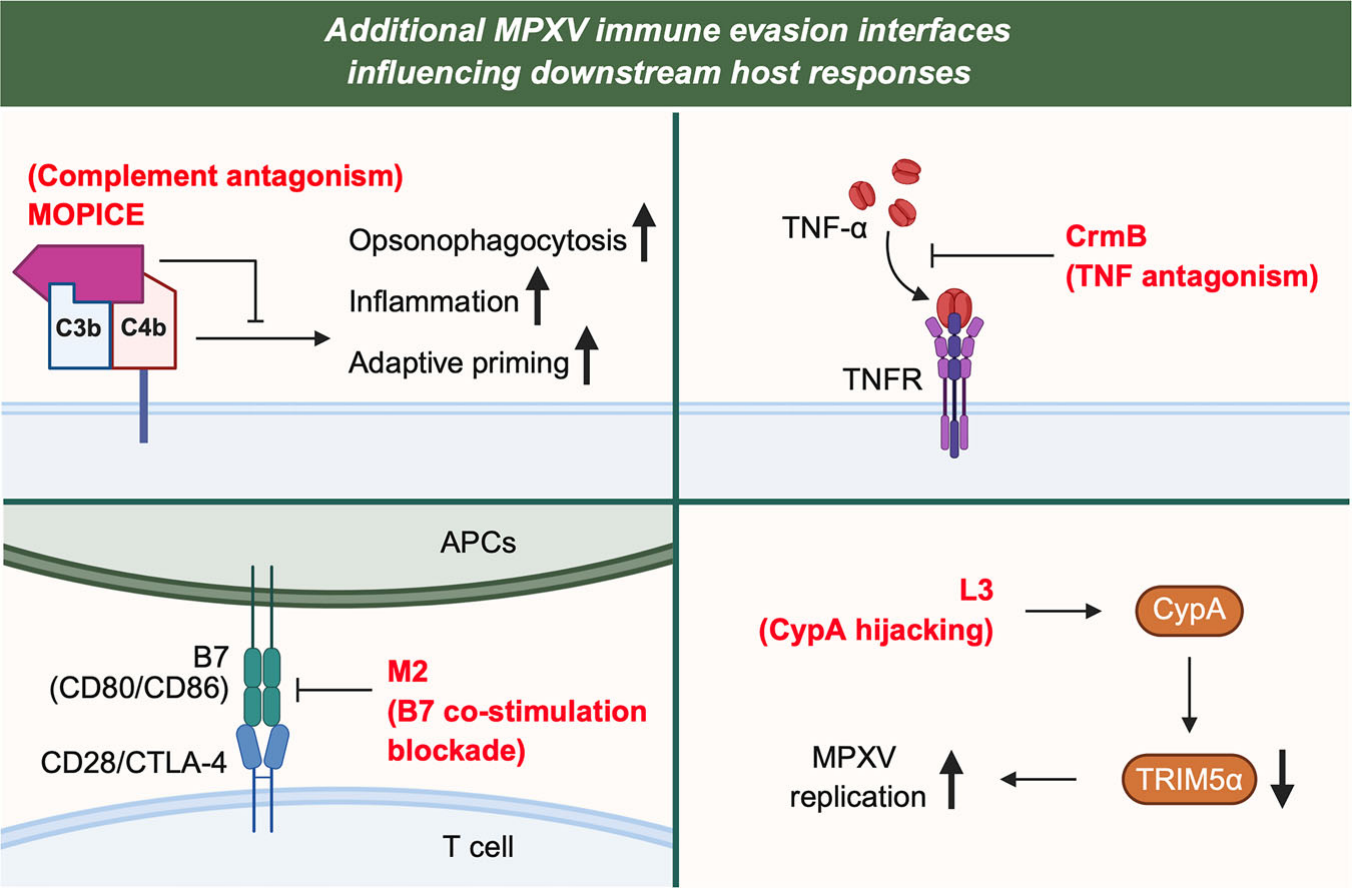
Additional MPXV immune evasion interfaces. MOPICE engages complement fragments C3b and C4b to suppress opsonophagocytosis, inflammation, and adaptive priming. CrmB antagonizes TNF-α signaling by blocking TNF-α engagement with TNFR; its contribution during authentic MPXV infection in vivo remains to be established. Viral L3 associates with host CypA, reducing TRIM5α abundance and thereby limiting TRIM5α-dependent restriction of MPXV replication. Secreted M2 interferes with B7 (CD80/CD86)-dependent co-stimulation of CD28 and CTLA-4 on T cells in APC-T cell synapses; direct evidence during authentic MPXV infection remains limited. APCs antigen-presenting cells, CrmB cytokine response modifier B, CTLA-4 cytotoxic T-lymphocyte-associated protein 4, CypA cyclophilin A, MOPICE monkeypox inhibitor of complement enzymes, MPXV monkeypox virus, TNF tumor necrosis factor, TNFR tumor necrosis factor receptor, TRIM5α tripartite motif-containing protein 5 alpha.Created in BioRender. Oh, J. (2026) https://BioRender.com/j6oxf91.

A study in VACV reported that a TRIM5α-L3-cyclophilin A (CypA) axis is antagonized by the VACV protein C6, which binds TRIM5α and induces its proteasome-dependent degradation. MPXV encodes a C6 homolog, and analogous L3 association with CypA and changes in cellular TRIM5α abundance were also observed in the context of MPXV infection^15^. Functionally, cyclosporine A and its derivatives (alisporivir and NIM811) have been reported to reduce MPXV plaque size and PFU-based titers, consistent with CypA-dependent restriction. However, whether the MPXV C6 homolog recapitulates the mechanistic role of VACV C6 in TRIM5α degradation, and the quantitative contributions of this axis during authentic MPXV infection, remain to be fully defined^15^ (Fig. 4).

MPXV encodes MOPICE, which is annotated as a complement control protein similar to host regulators of complement activation proteins^60–62^. This class of proteins has been shown to bind activated complement fragments such as C3b and C4b and dampen convertase-dependent amplification^60–62^. MOPICE status differs across MPXV clades, with retention more commonly described in Clade I genomes and deletions in Clade II lineages^60,62^. In vivo, recombinant MPXV lacking the complement control protein (CCP) in a Clade II showed reduced morbidity and mortality without a corresponding major reduction in the measured viral load^16^. Introducing CCP to a Clade I altered disease manifestations without an apparent mortality effect^16^. In a Clade I nonhuman primate study, the loss of MOPICE expression was associated with enhanced viral replication in vivo, together with a dampened adaptive immune response^17^. Collectively, these data indicate that MOPICE influences disease phenotypes in vivo but is not sufficient to account for broader clade-level differences^16,17,60–62^ (Fig. 4).

MPXV cytokine response modifier B (CrmB) antagonized TNF-mediated cytotoxicity in L929 cells, although no detectable survival benefit was observed in a lipopolysaccharide-induced endotoxic shock model. Its contribution to authentic MPXV infection in vivo remains to be established. The TNF-binding properties of CrmB differ across orthopoxviruses, with VARV CrmB showing a higher binding affinity for TNF than the MPXV and CPXV homologs, potentially contributing to the greater virulence historically attributed to VARV^18^ (Fig. 4). Secreted MPXV M2 binds human B7.1 and B7.2 and interferes with the engagement of CD28 and CTLA-4^19^. Cryo-EM and reconstituted human T cell activation experiments supported steric competition and reduced IL-2 output specifically under B7-dependent stimulation conditions^19^. However, direct evidence for this mechanism during authentic MPXV infection remains limited^19^ (Fig. 4).

### Immunopathogenesis and clinical correlates

#### Clinical manifestations

This section summarizes the outbreak-era clinical observations, which are organized from common manifestations to less frequent organ-specific complications. The evidence base is strongest for core mucocutaneous phenotypes, such as rash evolution, lymphadenopathy, proctitis, and ocular involvement, which recur across cohorts and systematic reviews. In contrast, cardiovascular, neurologic, and musculoskeletal complications are largely supported by case reports and small series and should currently be interpreted as uncommon events in selected contexts rather than defining mpox phenotypes.

Lymphadenopathy was one of the most consistently reported systemic findings in human mpox during a multi-country outbreak and was typically regional rather than generalized^63–65^. In a large U.S. jurisdictional surveillance analysis linked to clinical symptom reporting, lymphadenopathy was reported in approximately half of the cases, and its frequency differed modestly in relation to HIV status^66^. Specifically, individuals diagnosed with HIV infection were slightly less likely to report lymphadenopathy than those without HIV infection, whereas lymphadenopathy was more frequent among those with an unsuppressed HIV viral load within the subset with available HIV laboratory data^66^. Country-level cohorts have reported variable lymphadenopathy frequencies across settings, which likely reflect differences in ascertainment, case mix, and care pathways^63,64,67–69^. In systematic syntheses and narrative reviews of the 2022 outbreak and broader historical data, inguinal lymphadenopathy has been repeatedly highlighted as the most frequently characterized anatomical subtype of contemporary outbreak-associated diseases, which is consistent with the predominance of anogenital lesion presentations and related regional drainage patterns^67,70^.

**Fig. 5:**
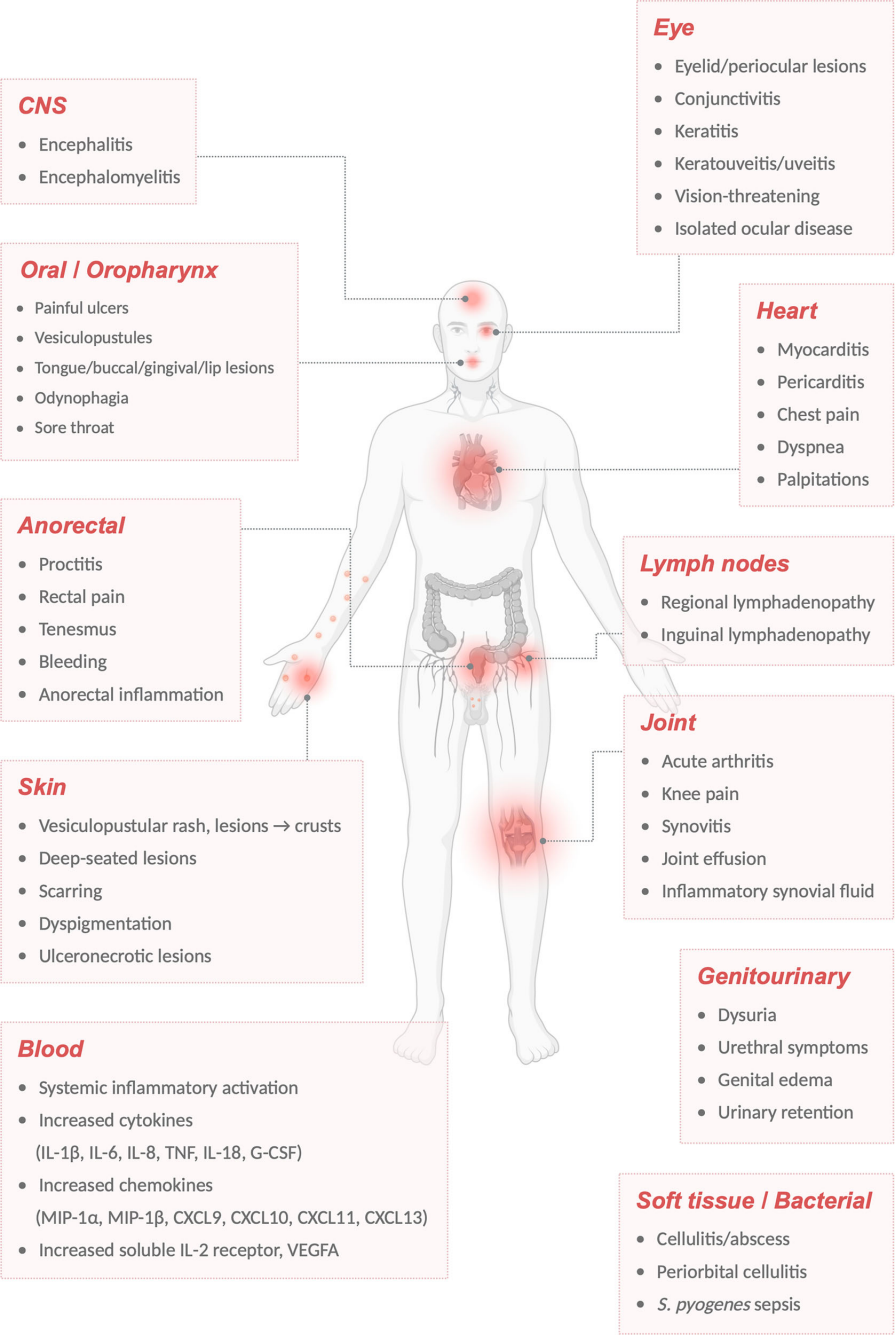
Clinical symptoms during MPXV infection. Human mpox typically presents as a multisite mucocutaneous disease and regional lymphadenopathy, with variable extracutaneous involvement. The key clinical sites include the skin, oral/oropharyngeal lesions, and anorectal disease. The reported extracutaneous phenotypes include ocular disease, cardiac involvement, joint involvement, genitourinary symptoms, and rare neurological complications. Clinical studies report elevations in the levels of circulating inflammatory mediators, and some cases show secondary bacterial soft-tissue complications. CNS central nervous system, CXCL C-X-C motif chemokine ligand, G-CSF granulocyte colony-stimulating factor, IL interleukin, MIP macrophage inflammatory protein, TNF tumor necrosis factor, VEGFA vascular endothelial growth factor A.Created in BioRender. Oh, J. (2026) https://BioRender.com/qgef8bf.

Rash was the defining clinical manifestation of human mpox in the 2022 outbreak, and was most commonly characterized by vesiculopustular lesions that progressed to crusting, with marked heterogeneity in lesion burden and anatomical distribution across patients^67,69,71–75^. Across dermatology-based case series and outbreak cohorts, lesions typically evolved in a time-linked manner from papules or vesicles to pustules and crusts, were deep-seated and well-circumscribed, and may have resulted in residual dyspigmentation or scarring in a subset of cases^67^. Histopathological studies showed viral cytopathic changes with epidermal and dermal inflammation, supporting clinically visible rash as tissue-level injury; severe disease may include extensive ulceronecrotic cutaneous involvement^76–79^ (Fig. 5).

Oral and oropharyngeal lesions have been reported to occur as part of the mucocutaneous spectrum of human mpox, with reviews, cohorts, and case series describing painful ulcerative or vesiculopustular involvement of the tongue, buccal mucosa, gingiva, lips, and oropharynx. This involvement is variably timed relative to cutaneous disease and may occasionally accompany anogenital involvement^65,72,73,80–82^. Across available human studies, oral symptoms, such as odynophagia or sore throat, were described as clinically relevant but generally self-limiting, whereas lesion-specific immune or virological correlates remained insufficiently characterized in cohort-level analyses^65,72,73,80–82^ (Fig. 5).

Proctitis is a recognized anorectal phenotype in human mpox outbreaks, supported by a systematic review and meta-analysis, a large multinational case series, and a prospective observational cohort collectively documenting anorectal symptoms within the clinical spectrum^65,67,83^. Imaging reports have provided objective correlates of anorectal inflammation consistent with rectal pain, tenesmus, and bleeding^84^, and the high viral loads detected in rectal samples further supported the direct mucosal involvement in mpox^85^. Although many cases are uncomplicated, case-based literature described severe presentations, including massive hematochezia or hemorrhagic proctocolitis, sometimes with prolonged rectal viral shedding and concurrent sexually-transmitted infections^86–92^ (Fig. 5).

Ocular involvement in human mpox has been reported as an uncommon but clinically important phenotype, and its presentations range from periocular or eyelid lesions and conjunctivitis to keratitis and keratouveitis or uveitis, with potential vision-threatening presentations described in outbreak literature^93–98^. Case-based evidence further shows that ocular mpox can present atypically, including isolated ocular disease without concurrent cutaneous lesions, and ocular involvement has also been reported in special populations, such as neonates, supporting meaningful clinical heterogeneity despite its low frequency^97,98^ (Fig. 5).

Mpox-associated myocarditis has been reported in immunocompetent adults presenting with acute chest pain and dyspnea, with overall favorable short-term outcomes based on the assembled case literature^99–101^. Pericardial involvement has also been described, including a case of mpox-associated pericarditis^102^. An updated cardiovascular review similarly highlighted chest pain, shortness of breath, and palpitations as common presenting symptoms across published cases of the cardiovascular manifestations of mpox^99,103^ (Fig. 5).

Acute arthritis during mpox has been reported to present with painful knee involvement and imaging findings such as synovitis and joint effusion, with inflammatory synovial fluid reported in at least one case report^104,105^ (Fig. 5). Neurological complications have been discussed in clinical overviews and reviews, including encephalitis/encephalomyelitis presentations during the 2022 outbreak^106–108^. In patients with advanced HIV infection showing severe necrotic and fatal mpox, mpox PCR positivity in the cerebrospinal fluid has been reported, whereas other case reports describing CNS involvement documented repeated cerebrospinal fluid (CSF) polymerase chain reaction (PCR) negativity, indicating heterogeneous CSF diagnostic findings across human cases^109–111^. A systematic review of neurological manifestations summarized the spectrum of reported neurologic outcomes across human studies while emphasizing the variability in ascertainment and case definitions across the included literature^112^ (Fig. 5). Genitourinary involvement has been summarized in urological reviews, and the manifestations include dysuria, urethral symptoms, genital edema, and urinary retention in selected patients^113–115^. A urology-focused perspective article likewise emphasized that genitourinary symptoms can be clinically relevant for specialty evaluation in mpox care pathways^114^ (Fig. 5). Cellulitis has been reported as a complication of mpox, with a two-patient report explicitly describing cellulitis as a complication and one case in which the initial presentation was periorbital cellulitis^116,117^. Clinical guidance documents also state that open lesions can progress to bacterial superinfections, resulting in cellulitis or abscess^118^. A case of severe mpox complicated by *Streptococcus pyogenes* sepsis was reported in a patient with HIV infection^119^. Evidence for these less common complications is mainly derived from case reports, small case series, and narrative or scoping syntheses and should be framed as uncommon complications reported in selected clinical contexts rather than defining mpox phenotypes (Fig. 5).

In an observational clinical immunology study, acute human mpox was accompanied by systemic symptoms and increased levels of circulating inflammatory mediators, including IL-1β, IL-6, IL-8, and TNF, which decreased over time but remained elevated after clinical recovery^5^. In a prospective hospitalized cohort with serial sampling, inflammatory profiling reported elevated levels of circulating mediators, including IL-6, IL-8, granulocyte colony-stimulating factor (G-CSF), macrophage inflammatory protein (MIP)-1α, and MIP-1β, supporting that measurable inflammatory signals track with clinically significant illness requiring inpatient care^120^. A large multiplex blood analysis of Clade IIb mpox patients quantified 65 cytokines, chemokines, and growth factors and found no distinct cluster-level profile that distinguished mpox patients from healthy controls. However, individual analyte analyses identified elevated levels of IL-18, soluble IL-2 receptor, CXCL9, CXCL10, CXCL11, CXCL13, and vascular endothelial growth factor A (VEGFA) in patients, indicating that selected immune mediators are quantifiably altered in the peripheral blood during clinical mpox, despite the absence of a defining composite signature^121^ (Fig. 5).

#### Association between the clinical outcomes of mpox and immune competence

Across cohorts, case series, and syntheses, moderate-to-severe immunocompromise has been repeatedly associated with severe human mpox, including prolonged illness, complications, and death, supporting risk stratification by functional immune status rather than diagnostic labels alone^122–124^. In immunocompromised states, human data distinguished controlled HIV infection from advanced or uncontrolled HIV infection in relation to mpox severity. In a global case series focused on advanced HIV infection, worse outcomes were clustered among individuals with lower CD4 counts and higher HIV viral loads, implying that the depth of immunosuppression was a major modifier of the disease trajectory^6^. Similarly, a large analysis reported an increased risk of mpox hospitalization among people with CD4 counts below 350 cells/μL, including those with suppressed viral loads or those who were out of HIV care, reinforcing that the depth of immunosuppression, rather than the HIV infection status alone, is a principal severity modifier^125^. Recent cohort and case-based reports further described severe presentations of advanced HIV infection, including multi-organ involvement, supporting the same severity gradient in independent clinical settings^126,127^. Severe mpox has also been reported in non-HIV-immunocompromised populations, including solid-organ transplant recipients and patients with malignancy or those undergoing immunosuppressive therapy. In a multicenter case series of solid-organ transplant recipients, hospitalization and serious complications, including death, were reported, supporting transplant status as a clinically relevant modifier of the disease course^128^. A systematic review and meta-analysis further supported higher hospitalization and mortality risks in immunosuppressed groups, while emphasizing heterogeneity across underlying conditions and care pathways when interpreting severity gradients^122^. Outside the immunocompromised categories, pediatric surveillance data from the United States and multi-country analyses indicated that cases were uncommon and overall mortality was low, although heterogeneity by age subgroup and exposure context was noted^129,130^. Case reports have described an extensive lesion burden in the setting of atopic dermatitis or eczema, supporting heightened clinical vigilance in selected barrier-disruption contexts^131,132^. Finally, across hospitalized cohorts and severe-manifestation summaries, escalation has been described in settings such as ocular involvement, neurologic disease, severe mucosal complications, extensive lesion burden, and secondary bacterial infection, indicating that the clinical context and timing of recognition and care can amplify disease severity beyond the baseline host status^123,133^. Narrative and topic-focused reviews of patients with HIV infection align with these signals and can be cited as synthesis support without changing the primary evidence hierarchy^134,135^.

Overall, functional immune competence is a major host-level modifier of human mpox outcomes. The strongest signal arises from advanced or uncontrolled immunosuppression, particularly advanced HIV infection, while non-HIV immunocompromised states also support risk stratification by the depth and durability of immune suppression.

### Therapeutic strategies

The therapeutic options for mpox remain anchored in repurposed orthopoxvirus countermeasures, and the interpretation of clinical benefits has evolved with the availability of randomized, large-scale clinical trial data for tecovirimat. In contemporary outbreak reports and clinical syntheses, tecovirimat is the most frequently deployed direct-acting antiviral, whereas cidofovir and brincidofovir are generally positioned as alternative or adjunct options in selected scenarios, given their toxicity constraints and limited mpox-specific human efficacy data^2,136^.

A large expanded-access analysis clarified that real-world treated cohorts were enriched for patients with severe immunocompromise and complicated disease courses, thereby constraining causal inference of efficacy. In the expanded-access analysis conducted in the U.S., tecovirimat was prescribed at scale, and serious adverse events were not commonly reported, but life-threatening or protracted infections were concentrated in severely immunocompromised patients. The authors emphasized that controlled trial data are required to determine whether and how tecovirimat should be used^137^.

Randomized evidence provides the clearest recent anchor for benefit assessment. In the PALM007 double-blind, randomized, placebo-controlled trial in the Democratic Republic of Congo evaluating tecovirimat for Clade I mpox, the primary endpoint of time-to-lesion resolution did not differ between groups. The estimated median was 7 days with tecovirimat versus 8 days with placebo, with a stratified competing-risks hazard ratio of 1.13 and a 95% confidence interval of 0.97 to 1.31. Safety signals were not meaningfully different between the groups, supporting tolerability while not demonstrating a clinical benefit for lesion resolution^138^.

Beyond tecovirimat, nucleotide analog-based agents represent an additional therapeutic strategy that is supported primarily by preclinical data and limited clinical experience. Contemporary reviews typically frame cidofovir and brincidofovir as candidates in selected scenarios, while emphasizing the limited mpox-specific human efficacy data and well-recognized toxicity constraints that narrow practical use^2,136^. In controlled preclinical studies, these agents have shown antiviral activity against MPXV in vivo, supporting continued evaluation as a complementary option when the therapeutic benefit is uncertain or when resistance is a concern^139^.

Antiviral resistance is the second theme that can materially reshape the therapeutic landscape, particularly since viral replication can persist for prolonged periods after tecovirimat exposure. Genomic surveillance of clinical specimens has identified recurrent amino acid substitutions in the tecovirimat target gene *F13*, including both previously described resistance-associated mutations and novel candidate variants, supporting the value of integrating viral genomics into treated cohorts^140–142^. In an outbreak-era clinical series, severe immunocompromise was associated with protracted disease courses and repeated antiviral exposure, providing a practical context in which resistance evaluation and genomics-informed trial stratification may be most relevant^6^. Recent structural studies have mapped clinically observed resistance-associated changes to the *F13* target in a mechanistic model, strengthening the interpretation of sequence variation alongside clinical outcomes^143^.

Emerging approaches include host modulation of inflammatory cell death, structure-guided inhibition of viral immune modulators such as H1 phosphatase, and antigen-defined neutralizing monoclonal antibodies. These strategies remain preclinical or early translational, as their efficacy has not yet been established in mpox-specific human studies^52,144–148^.

Overall, tecovirimat remains widely used owing to its acceptable safety profile, but randomized trials have not shown improved lesion resolution, and observational data are confounded by prioritization of severely immunocompromised patients. Nucleotide analogs remain limited by toxicity and insufficient mpox-specific human efficacy data. Prolonged replication and repeated antiviral exposure highlight the need for genomics-integrated monitoring and trial stratification.

### Conclusions and future perspectives

Recent studies indicate that early host responses contribute to mpox severity. MPXV activates DNA sensors, including cGAS and AIM2, which drive distinct outcomes such as interferon induction and inflammatory cell death, although their hierarchy and timing remain incompletely defined. AIM2 links MPXV DNA detection to caspase-1 activation, pyroptosis, and IL-1 family cytokine release. Genetic or pharmacological AIM2 inhibition reduces inflammation and tissue pathology but may increase viral dissemination, indicating a context-dependent balance between viral restriction and immunopathology^8^. In parallel, MPXV activates cGAS-STING signaling but often induces weak endogenous interferon responses in productive human cell infection, consistent with viral evasion by P2, OPG147, interferon-binding proteins, and PoxS^11–14^. These mechanisms may contribute to an imbalance between IFN-linked restriction and inflammasome-linked inflammation while delaying effective antiviral restriction. The balance between IFN-driven control and inflammasome-driven pathology is influenced by viral evasion, tissue context, and host immune competence. Experimental animal data suggest that MPXV may access the CNS via two routes: the olfactory epithelium and hematogenous spread by infected circulating monocytes/macrophages that cross the blood-brain barrier^107^. In the CNS, MPXV-infected human astrocytes undergo inflammatory cell death via GSDMB rather than GSDMD, suggesting that execution pathways differ across tissues and species^52^. These observations have not been reproduced in standard mouse models and limit cross-species extrapolation. The host immune status critically influences outcomes. People with advanced HIV infection, particularly those with CD4 counts < 200 cells/μL, are disproportionately affected by severe or disseminated mpox. This disease involves ulceronecrotic skin lesions, pulmonary and gastrointestinal complications, and persistent viremia^6^. Transcriptomic profiling in people living with HIV infection indicated attenuated Type I IFN and ISGs programs in peripheral immune compartments during mpox^149^. At the tissue level, skin biopsy analysis of prolonged mpox cases in people with advanced HIV revealed absent or markedly reduced Langerhans cells at lesion sites, alongside scarce CD4 + T cells and absent IFN-γ, despite robust innate immune infiltration, indicating insufficient local adaptive immunity to control viral replication^150^. Separately, a large clinical series of mpox in cases with advanced HIV infection documented frequent disseminated disease with high viral burden and severe outcomes^6^. Together, these reports indicate that impaired IFN-linked antiviral programs coexist with systemic viral spread in vulnerable hosts, although a direct causal linkage within a single study has not yet been established^6,149^.

Immune reconstitution following antiretroviral therapy initiation has been associated with immune reconstitution inflammatory syndrome (IRIS)-like syndromes involving worsening mpox inflammation in individual case reports^6,151^. However, a retrospective observational study comparing early and late ART initiation found no worse outcomes associated with mpox-related IRIS among patients who initiated ART early, suggesting that prompt ART initiation remains appropriate in the absence of contraindications^152^. Recent reports have described the re-emergence of type 1 cytokine networks during immune restoration, whereas autopsy studies of fatal immunocompromised mpox documented widespread tissue viral burden with impaired IFN-γ programs in lymphoid organs^153^. In contrast, individuals with well-controlled HIV infections or normal CD4 counts showed outcomes comparable to those in HIV-negative populations^154^.

In solid-organ transplant recipients, a multicenter clinical series described mpox presentations under chronic immunosuppression and reported clinically significant morbidity requiring antiviral therapy and tailored immunosuppression management^128^. In broader cohorts that included patients receiving immunosuppressive therapies, immunosuppression was associated with an increased risk of hospitalization, including those treated for malignancy^155^. As cancer-specific mpox cohorts remain fragmented across reports, these data are best cited as convergent evidence rather than as a single unified malignancy cohort analysis^128,155^.

These clinical and virological insights have translational implications in clinical practice. Exogenous Type I IFN therapy has demonstrated efficacy in animal models by reducing viral replication and systemic inflammation^7^. Conversely, AIM2-targeted modulation has been shown to attenuate IL-1β and IL-18 release, alleviating tissue damage in susceptible hosts^8^. Consequently, combining antiviral strategies with immunomodulatory approaches may be beneficial, particularly for immunocompromised patients. However, indiscriminate immunosuppression may permit viral dissemination, underscoring the need for careful therapeutic calibration^153^.

Preclinical models show several limitations. Common mouse strains do not replicate the susceptibility seen in CAST/EiJ mice, which lack robust early IFN-γ responses and develop severe mpox with systemic spread^53,57^. However, even the susceptible CAST/EiJ strains fail to capture the full complexity of HIV co-infection. Notably, human-relevant features, such as GSDMB-mediated pyroptosis or MPXV immune evasion of STING, may be absent or functionally divergent in murine systems. These species-specific discrepancies highlight the danger of extrapolation across systems^156^. This limitation directly affects the interpretation of mechanistic and therapeutic findings derived from murine models. Therefore, representative models incorporating immunodeficiency, tissue diversity, and human immune components are required. Overall, mpox immunopathogenesis reflects the interactions among viral DNA sensing, immune evasion, and host immune competence. The existing evidence delineates parallel IFN and inflammasome pathways, but it is limited by the use of fragmented clinical cohorts, incomplete temporal mapping of sensor activation, and species-restricted model systems. Future studies should integrate data from longitudinal human studies, tissue-resolved immune profiling, and mechanistically aligned preclinical models to clarify the pathway hierarchy and develop targeted intervention strategies, particularly for immunosuppressed populations^153^.
